# Hsa_Circ_0066351 Acts as a Prognostic and Immunotherapeutic Biomarker in Colorectal Cancer

**DOI:** 10.3389/fimmu.2022.927811

**Published:** 2022-07-13

**Authors:** Yan Gao, Yulai Zhou, Le Wei, Ziyang Feng, Yihong Chen, Ping Liu, Yinghui Peng, Qiaoqiao Huang, Le Gao, Yongting Liu, Ying Han, Hong Shen, Changjing Cai, Shan Zeng

**Affiliations:** ^1^ Department of Oncology, Xiangya Hospital, Central South University, Changsha, China; ^2^ Key Laboratory for Molecular Radiation Oncology of Hunan Province, Xiangya Hospital, Central South University, Changsha, China; ^3^ National Clinical Research Center for Geriatric Disorders, Xiangya Hospital, Central South University, Changsha, China

**Keywords:** ceRNA, colorectal cancer, hsa_circ_0066351, biomarker, immunotherapy

## Abstract

Circular RNA (circRNA), a novel class of non-coding RNA, has been reported in various diseases, especially in tumors. However, the key signatures of circRNA-competitive endogenous RNA (ceRNA) network are largely unclear in colorectal cancer (CRC). We first characterized circRNAs profile by using circRNA-seq analysis from real-word dataset. The expression level of hsa_circ_0066351 in CRC tissues and cell lines was detected by quantitative real-time PCR. Then, cell proliferation assay was used to confirm the proliferation function of hsa_circ_0066351. Next, Cytoscape was used to construct circRNA–miRNA–mRNA networks. Last but not least, the landscape of hsa_circ_0066351–miRNA–mRNA in CRC had been investigated in the bulk tissue RNA-Seq level and single-cell Seq level. We proved that hsa_circ_0066351 was significantly downregulated in CRC cell lines and tissues (P < 0.001), and was negatively associated with distant metastasis (P < 0.01). Significantly, the expression of hsa_circ_0066351 was associated with better survival in patients with CRC. Function assays showed that hsa_circ_0066351 could inhibit CRC cells proliferation. In addition, a ceRNA network, including hsa_circ_0066351, two miRNAs, and ten mRNAs, was constructed. Our analyses showed that these ten mRNAs were consistently downregulated in pan-cancer and enriched in tumor suppressive function. A risk score model constructed by these ten downstream genes also indicated that they were related to the prognosis and immune response in CRC. In conclusion, we demonstrated that a novel circRNA (hsa_circ_0066351) inhibited CRC proliferation, and revealed a potential prognostic and immunotherapeutic biomarker in CRC.

## Introduction

Colorectal cancer (CRC) is one of the most common malignant tumors in the world, ranking as the third threat to cancer-related death (1). Worryingly, approximately 25% of CRC patients are diagnosed at a terminal stage and untreatable by surgery, calling for more accurate early diagnosis and individualized treatment (2, 3). Based on this situation, a large number of clinical trials and applications of immunotherapy have been carried out in patients with CRC (4). Thus, it is urgent to develop biomarkers for early diagnosis and immunotherapy of CRC to improve the prognosis of patients.

Circular RNAs (circRNAs) are identified as a new class of endogenous non-coding RNAs (ncRNAs) formed by back splicing of precursor mRNA transcripts and distinguished by a covalently closed ring structure (5–7). In recent years, with the application of high-throughput sequencing technology (8), a great number of circRNAs have been discovered, which show that circRNAs are structurally stable and play key roles in the pathophysiologic mechanism of several diseases, especially malignant tumors (9, 10). MicroRNAs (miRNAs) have been found to act as cancer suppressor genes and oncogenes in diverse tumors (11). An increasing number of research have indicated that circRNAs are involved in post-transcriptional regulation by functioning as the competitive endogenous RNAs (ceRNAs) of miRNAs (12, 13). It has been validated that circRNAs are involved in the tumorigenesis of CRC and could serve as a potential biomarker (14). For instance, circHERC4 could inhibit the progression of CRC *via* the miR-556-5p/CTBP2/E-cadherin pathway (15). CircPLCE1 was downregulated in CRC and could improve progression by promoting RPS3 ubiquitin-dependent degradation (16). Furthermore, circRNAs were able to interact with proteins and participate in protein translation (14, 17). However, the specific mechanism and role of circRNAs in the tumorigenesis and progression of CRC remain inadequate. Therefore, it is of great significance to further explore circRNAs as a novel biomarker and therapeutic target for CRC.

Recently, several researches have shown that immune cells in tumor microenvironment (TME) play an important role in tumor immune escape and immunotherapy resistance (18, 19). As previous studies were focused on only one type of immune cell and one gene, the understanding of the CRC microenvironment may be incomplete (20, 21). Thus, the construction of a gene signature that integrates the role of multiple key genes could improve our understanding of TME features and identify novel biomarkers to screen patients for treatment with immune checkpoint inhibitors.

In this study, we explored the expression difference of circRNAs in CRC patients through sequencing to find functional circRNAs. Hsa_circ_0066351 was found to be significantly downregulated in CRC samples and related to the proliferation and prognosis of CRC patients. Meanwhile, we also revealed the significance of the potential ceRNA network regulated by hsa_circ_0066351 and established a risk score model for target mRNAs. Therefore, our findings indicate that hsa_circ_0066351 and its regulated ceRNA network may serve as a prognostic and immunotherapeutic biomarker in CRC.

## Materials and Methods

### Clinical Samples

CRC tissues and their matching adjacent normal tissues were collected from 47 CRC patients who underwent radical resection (without preoperative adjuvant therapy) in Xiangya Hospital of Central South University. CRC patients were diagnosed by histopathology or tissue biopsy. The detailed clinicopathological features of these samples are described in **Table 1**. Tumor staging was based on the Union for International Cancer Control (UICC) CRC tumor lymph node metastasis (TNM) classification in 2003, and tumor grading was based on the World Health Organization (WHO) 2010 classification protocol. This study was approved by the Clinical Research Ethics Committee of Xiangya Hospital, Central South University.

**Table 1:** Clinical characteristics of CRC patients with low hsa_circ_0066351 expression and high hsa_circ_0066351 expression.

Clinical feature	Group	Cases	Hsa_Circ_0066351 expression		
			Low	High	*P*-value
Age	≤53	16	9	7	0.1724
	>53	31	11	20	
Gender	Male	27	11	16	0.9949
	Female	20	9	11	
T grade	T1 + T2	4	2	2	0.7528
	T3 + T4	43	18	25	
Tumor size	≤5cm	23	10	13	0.9001
	>5cm	24	10	14	
Stage	I-II	20	10	10	0.3742
	III-IV	27	10	17	
Distant metastasis	M0	33	9	24	0.028^*^
	M1	14	11	3	
Lymphatic invasion	Negative(N0)	25	8	17	0.1188
	Positive(N1-N3)	22	12	10	

The bold values mean that the P value of the values are less than 0.05, which is statistically significant.

### Cell lines and Cell Culture

CRC cell lines (LoVo, Caco2, HCT8, HCT116, SW480, SW620) and healthy fetal human colon (FHC) cells were purchased from the Institutes of Biomedical Sciences (IBS, Shanghai, China). Cells are stored and disposed of according to the provider’s instructions. LoVo, Caco2 and FHC cells were kept in a MEM Medium (Gibco, USA). SW480 and SW620 were kept in L-15 Medium (Gibco, USA). HCT116 and HCT8 were kept in RPMI‐1640 Medium (Gibco, USA). The medium was supplemented with 10% fetal bovine serum (Gibco, USA) and 1% penicillin and streptomycin (Gibco, USA). All cell lines were cultured in a humidified incubator at 37°C with 5% CO_2_.

### Quantitative Real-Time Reverse Transcription Polymerase Chain Reaction

TRIzol reagent (Invitrogen, Carlsbad, CA) was used to extract total RNA and amplify cDNA according to the manufacturer’s instructions with Prime Script Kit (TaKaRa Bio Inc., Otsu, Japan) (1 ug per sample). RT-qPCR with three replicates was performed using SYBR green fluorescence assay (638320, TaKaRa Bio Inc.) on the ViiATM7 RT-qPCR system (Applied Biosystems, Carlsbad, CA). The primers for RT-qPCR were as follows: FLNB forward: 5′-ACTGTCATGGCCACAGATGG-3′; reverse: 5′-AAATCCCAGGCCGTTCATGT-3′; GAPDH forward: 5′-CGGAGTCAACGGATTTGGTCGTAT-3′; reverse: 5′-AGCCTTCTCCATGGTGGTGAAGAC-3′; hsa_circ_0066351 forward: 5′-GAGGCCGATGTCATTGAGAA-3’; reverse: 5’-GCTCCACCAAGCCATGACAG-3’; Relative mRNA expression levels were calculated by the 2−(ΔΔCt) method and were normalized to the internal control (GAPDH), ΔCt = Ct (targeting gene) − Ct (GAPDH), and ΔΔCt =ΔCt (treated) – ΔCt (control).

### Identification of Hsa_Circ_0066351

The backsplicing sequence of hsa_circ_0066351 was verified through circBase database (22). Amplification products of hsa_circ_0066351 were confirmed by Sanger sequencing from Geneseed Biotech (Guangzhou, China).

### RNase R Treatment

RNase R (Geneseed Biotech, Guangzhou, China) was used to digest linear RNA. RNA molecules extracted from CaCO_2_ and HCT116 cells were divided into two groups: one group was treated with RNase R and the other group was a control group. Both groups were incubated with 2.5 U/μg RNase R at 37°C for 30 min. Afterwards, hsa_circ_0066351 and linear FLNB were detected using reverse transcription and RT‐qPCR. The control group took GAPDH as the internal reference. Three separate experiments were repeated three times.

### Virus Transfection

A lentiviral vector pHBLV-CMV-MCS-EF1-Zsgreen1-T2A-puro containing human circ_0066351 was constructed by Hanbio Biotechnology (Shanghai, China). ShRNA targeting hsa_circ_0066351 or random sequence was synthesized and inserted into the lentiviral vector pHBLV-U6-MCS-CMV-ZsGreen-PGK-PURO of Hanbio Biotechnology (Shanghai, China). CRC cells were then transduced with appropriate lentiviruses. To screen stable transduction cells, puromycin (2 μg/mL) was resuspended for 2 weeks. The level of hsa_circ_0066351 was detected by quantitative reverse transcription-polymerase chain reaction (RT-qPCR).

### Cell Counting Kit−8 Proliferation Assay

Proliferation of CRC cells was detected by CCK-8 assay (APExBIO, Houston, USA). The cells at the logarithmic growth stage (1000 cells per well) were inoculated in 96-well plates. At the specified time (24, 48, 72 and 96 h), 10 μL of cell counting Kit 8 (CCK-8) solution was added to each well and incubated at 37°C for 2 h. The absorbance at 450 nm was calculated by spectrophotometry 3 times every 24 h.

### Colony Formation Assay

One thousand CRC cells were seeded in each well of the 6‐well plates and incubated at 37°C for 10 days. After removing the medium, the cells were washed with PBS (phosphate-buffered saline), fixed with 4% paraformaldehyde for 10 min, and stained with 0.5% crystal violet solution for 20 min. The numbers of colonies were counted and examined. The assay was carried out three times.

### 5‐Ethynyl‐2’‐Deoxyuridine Incorporation Assay

EdU assays were performed using the Cell-light EdU DNA Cell Proliferation Kit (RiboBio, Guangzhou, China) according to the manufacturer’s protocol. The cells (3 × 10^4^) were implanted into each well of the 96-well plate and cultured for 24 h to put them in the logarithmic growth phase. After 2 h culture with 50 μM EdU, the cells were fixed with 4% paraformaldehyde and stained with Apollo Dye Solution. Nucleic acids were stained with Hoechst 33342. EdU-positive cells were then photographed and IPP software was used to analyze the obtained data. The assay was carried out three times.

### Identification of Differentially Expressed MiRNAs and Analysis of Target MiRNAs

We identified differentially expressed (DE) miRNAs in TCAG COAD cohort and analyzed the expression, hazard ratio, and the area under the receiver operating characteristic (ROC) curve (AUC) of the target miRNAs in pan-cancer by CancerMIRNome (http://bioinfo.jialab-ucr.org/CancerMIRNome/) (23).

### Analysis of CircRNA-MiRNA-mRNA Network

The target miRNA binding sites were predicted by online websites: Circbank, circMine, and Starbase (24–26). For hsa_circ_0066351, we showed 3 common miRNAs combined prediction results with TCGA-upregulated DEmiRNAs. Target mRNAs were predicted by Targetscan (www.targetscan.org), miRDB (www.mirdb.org) and miRTarBase (27). After taking the intersection between TCGA-downregulated DEmRNAs and predicted mRNAs, we identified 13 possible downstream genes regulated by hsa-miR-27a-3p and hsa-miR-379-5p. Cytoscape software was applied to construct a circRNA-miRNA-mRNA network map (28).

### Identification of Key Genes

The mRNA expression data for CRC were obtained from The Cancer Genome Atlas (TCGA) database (https://portal.gdc.cancer.gov/). The colon cancer mutation data and clinical information were also downloaded from TCGA database. DEmRNAs were screened in CRC patients and healthy individuals by using the Bioconductor Limma package (version 3.50.1) (29). The thresholds of adjusted p < 0.05 and |log FC| > 1 were used to identify DE genes (DEGs) from TCGA colorectal cancer datasets. Based on the clinical information from TCGA, we used the MaxStat R package to determine the optimal cut-off point to classify patients into high and low expression groups. We performed KM curve to identify the prognosis of DEGs. *P* < 0.05 as significant.

### Pan-Cancer Analysis and Drug Sensitivity for Target Genes

GSCALite (http://bioinfo.life.hust.edu.cn/web/GSCALite/), a website that provides cancer gene expression, single nucleotide mutation, methylation and cancer pathways of active data, was used to analyze the expression, methylation, mutation, pathway activities, and drug sensitivity of target mRNAs (30).

### Functional Enrichment of Target Genes Based on Single Cell Data

CancerSEA (http://biocc.hrbmu.edu.cn/CancerSEA/home.jsp) is a multi-functional website designed to comprehensively explore the different functional states of cancer cells at the single-cell level, covering 14 cell functional states and 900 single cells of 25 cancer types (31). The CancerSEA database was used to analyze 10 key downstream genes function in CRC using cancer single cell data.

### Association with TME Infiltration Cells

The abundance of cell infiltration in TME was quantified using single-sample gene-set enrichment analysis (ssGSEA) based on the gene sets obtained from CIBERSORT and Charoentong’s study (32, 33). We tried to make up for the bias due to tumor purity by adjusting the enrichment fraction of each TME cell subtype by estimating tumor purity using the ESTIMATE algorithm (34). The abundance of each TME infiltration cell was represented by the adjusted enrichment fraction calculated by ssGSEA analysis. A total of 24 human TME cell subtypes were evaluated.

### Construction of Prognostic Signatures Based on Key Genes

We constructed riskScore signatures based on the overall effect of these 10 genes in COAD progression to comprehensively analyze the role of these genes in tumor patient prognosis, immune cell infiltration and immunotherapy effect. The Least Absolute Shrinkage and Selection Operator (LASSO) Cox regression was used to reduce the dimensions and the most secure markers were used to construct the riskScore signature (35), and the accuracy was assessed using receiver operating characteristic (ROC) analysis. The riskScore signature was defined as: $risk\;score=\sum_{i=1}^{n}Coefi*Expri$ , where Coefi represents the coefficient obtained by LASSO Cox regression and Expri represents the expression level of key genes. The risk factor of patients was calculated by this signature.

### Functional Annotation for Key Genes From High and Low Riskscore Groups

R package “TCGAbiolinks” was used to classify patients into high-risk and low-risk groups (29, 36). We performed gene ontology (GO) and KEGG enrichment analysis using clusterProfiler R packages to explore the potential biological functions of key genes (29). Finally, we visualized the results obtained by “ggplot2” R package.

### Statistical Analysis

The difference between the two groups was analyzed by Wilcoxon test. The correlation test is based on Spearman correlation analysis. The statistically significant difference between three or more groups was tested by One-way ANOVA and Kruskal-Wallis tests (29). Hazard ratio (HR) and 95% confidence interval (CI) were calculated using a univariate Cox regression model. The value of riskScore as an independent prognostic biomarker was assessed using a multivariate Cox regression model. The “maftools” R package was used to explore mutations in key genes in CRC patients (37). All statistical P values were bilateral, p < 0.05 was considered statistically significant. All data were processed by R4.1.1 software.

## Results

### Hsa_Circ_0066351 Was Downregulated in CRC Tissues and Cell Lines

**Figure 1:**
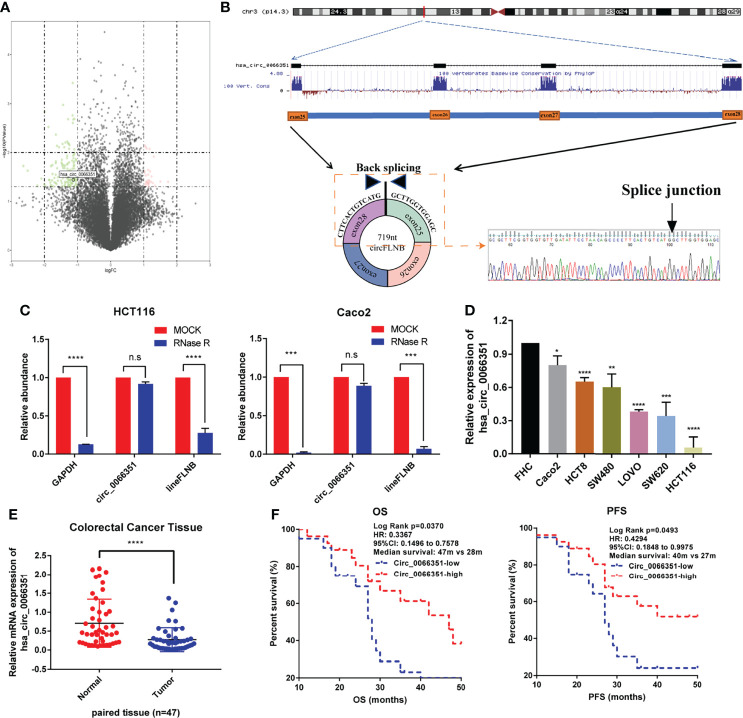
Identification and characterization of Hsa_circ_0066351 in CRC cells and tissues. **(A)** Volcano map showing the differences in the expression of circRNAs in CRC tissues and adjacent normal tissues. **(B)** Explanation of the illustrated genomic loci of FLNB, and the verification strategy for the circular exon 25–28 (hsa_circ_0066351). Sanger sequencing following PCR was used to show the “head-to-tail” splicing of hsa_circ_0066351. **(C)** Relative RNA level of hsa_circ_0066351 and linear FLNB treated with RNase R. **(D)** Different expression levels of hsa_circ_0066351 in CRC cell lines. **(E)** RT-qPCR shows that the expression of hsa_circ_0066351 in CRC tissues was significantly lower than that in adjacent normal tissues. **(F)** The expression of hsa_circ_0066351 was validated in a subset of 47 pairs of CRC and adjacent non-tumorous tissues. OS and PFS analyses were performed by the Kaplan–Meier test and the log-rank method in the cohort. All the data are presented as the mean ± SEM. *P < 0.05, **P < 0.01, ***P < 0.001, ****P< 0.0001. ns, not significant.

We first characterized circRNAs profile by using circRNA-seq analysis from three pairs of human CRC tissues and adjacent normal tissues. The samples used for analyses came from the Department of Oncology of Xiangya Hospital. According to sequencing reports of significant differences in log2FC expression levels of RNAs, we detected 13206 distinct circRNAs in total and 158 differentially expressed circRNAs, among which 30 circRNAs were upregulated and 128 circRNAs were downregulated in CRCs when compared with adjacent normal tissues. According to the experimental results of differential expression in human CRC tissues and adjacent normal tissues, we selected the circRNA (has_circ_0066351) which has the most significant difference as the next research target (**Figure 1A**).

Hsa_circ_0066351, derived from the FLNB gene on chromosome 3, consists of exon25, exon26, exon27 and exon28 spliced head to tail. Sanger sequencing result showed that the backsplicing sequence of hsa_circ_0066351 was consistent with that of circBase (**Figure 1B**). HCT116 and Caco2 cells were treated with RNase R respectively to identify the stability of hsa_circ_0066351. RT‐qPCR was used to detect the relative expression levels of hsa_circ_0066351 and linear FLNB to analyze their stability. The results obtained show that hsa_circ_0066351 was more stable than linear FLNB (**Figure 1C**). Then, we detected the expression levels of hsa_circ_0066351 in 47 frozen CRC tissues along with adjacent normal tissues through RT‐qPCR. The results obtained validate that hsa_circ_0066351 expression was significantly downregulated in 47 pairs of colorectal cancer tissues when compared with normal tissues, which is consistent with our previous analysis (**Figures 1D, E**).

Clinical data from 47 patients with colon cancer in Xiangya Hospital showed that decreased hsa_circ_0066351 expression level was associated with distant metastasis (**Table 1**). Furthermore, the survival information of these patients was collected and the curves of overall survival (OS) and progression-free survival (PFS) were plotted by Kaplan Meier method. Patients with higher expression level of hsa_circ_0001666 in CRC had higher OS rate (*p* = 0.013) and PFS rate (*p* = 0.023) (**Figure 1F**). Taken together, these results suggested that hsa_circ_0066351 was significantly downregulated in CRC and higher hsa_circ_0066351 expression may indicate a better prognosis.

### Hsa_Circ_0066351 Suppressed the Proliferation of CRC Cells *In Vitro*

**Figure 2:**
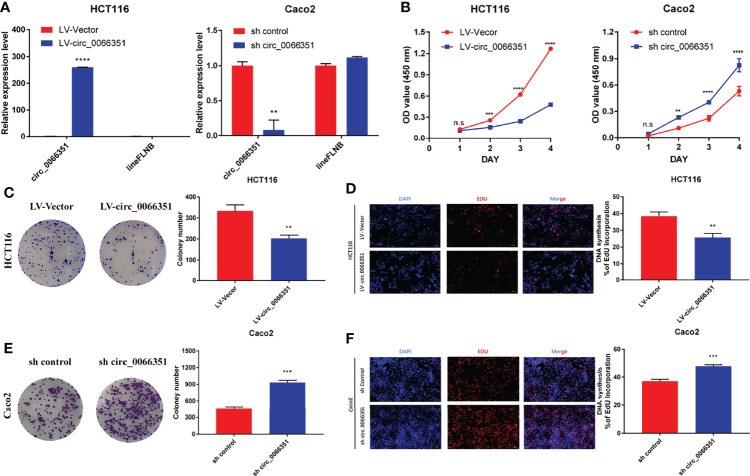
Hsa_Circ_0066351 inhibits cancer proliferation of CRC cells. Experiments were conducted after transfecting HCT116 and Caco2 cells with lentivirus. **(A)** Hsa_circ_0066351 was overexpressed and knocked down in HCT116 and Caco2 cells respectively, which was analyzed by RT-qPCR. **(B)** Cell Counting Kit-8 (CCK-8) assays were used to determine the effect of hsa_circ_0066351 on cell viability. **(C, D)** The effect of hsa_circ_0066351 on colony formation was determined *via* a clonogenicity assay. **(E, F)** EdU assays showed that overexpression of hsa_circ_0066351 inhibited the proliferation of CRC cells, while knockdown of hsa_circ_0066351 promoted this process; EdU scale bar=100 μm. Error bars represent the mean ± SEM from three independent experiments. *P < 0.05, **P< 0.01, ***P< 0.001, ****P< 0.0001. ns, not significant.

To determine the functional role of hsa_circ_0066351, we selected HCT116 cells and CaCO_2_ cells for functional study based on hsa_circ_0066351 expression in CRC cell lines. The increase and decrease of hsa_circ_0066351 expressions induced by lentivirus were confirmed by RT-qPCR analysis in HCT16 and Caco2 cells, respectively (**Figure 2A**).

CCK-8 assays confirmed that the overexpression of hsa_circ_0066351 significantly inhibited the proliferative activity of cells, while the knockdown of hsa_circ_0066351 showed an opposite effect (**Figure 2B**). What’s more, colony formation assays also showed that the overexpression of hsa_circ_0066351 significantly inhibited the cloning ability of HCT116 cells, whereas the knockdown of hsa_circ_0066351 greatly increased this ability in Caco2 cells (**Figures 2C, E**). EdU assays further demonstrated that the cell proliferation capabilities of HCT116 were obviously impaired by the upregulation of hsa_circ_0066351 and markedly enhanced by the downregulation of hsa_circ_0066351 (**Figures 2D, F**). All these data indicate that hsa_circ_0066351 could inhibit the proliferation of CRC cells.

### Construction of a CeRNA Network

**Figure 3:**
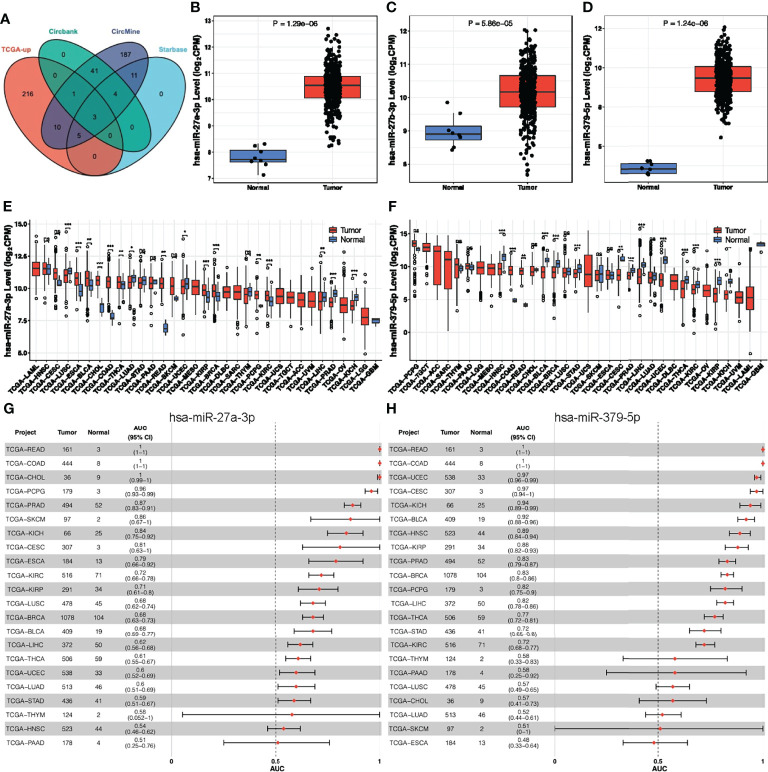
Analysis of hsa_circ_0066351 targeted miRNAs in COAD and pan-cancer. **(A)** The Venn diagram of predict targeted miRNAs from TCGA DEmiRNAs and three datasets. **(B)** miR-27a-3p expression in TCGA-COAD. **(C)** miR-27b-3p expression in TCGA-COAD. **(D)** miR-379-5p expression in TCGA-COAD. **(E)** miR-27a-3p expression in TCGA pan-cancer. **(F)** miR-379-5p expression in TCGA pan-cancer. **(G)** Forest plot visualizing miR-27a-3p in pan-cancer AUC analysis. **(H)** Forest plot visualizing miR-379-5p in pan-cancer AUC analysis. *P < 0.05; **P < 0.01; ***P < 0.001; ns, not significant.

As previous studies have shown that circRNAs primarily acts as a sponge for microRNAs (miRNAs) (38, 39), we predicted possible miRNAs targeted by hsa_circ_0066351 from three online databases (circBank, circMine, and StarBase). Combined with TCGA-upregulated differentially expressed miRNAs, we screened out three miRNAs that overlapped (**Figure 3A** and **Supplementary Figure 1A**). And we validated the expression of three miRNAs in TCGA (**Figures 3B–D**). Next, we utilized three databases (Targetscan, miRDB and miRTarBase) to predict the target genes of overlapped miRNAs (**Supplementary Figures 1B–D**). Combined with TCGA-downregulated differentially expressed mRNAs, we finally screened out 13 target genes (**Supplementary Figures 1E, F**). According to the ceRNA theory, we finally identified 2 miRNAs (miR-27a-3p and miR-379-5p) and 13 mRNAs (MIER3, NEURL1B, LITAF, PRKAA2, GNG12, CCNYL1, LIFR, SEMA6A, TGFBR3, PHLPP2, MAP1B, THRB, and SLC20A1) as hsa_circ_0066351 regulated ceRNA network, and the binding sites of has_circ_0066351 with miR-27a-3p and miR-379-5p were showed in **Supplementary Figure 1G**. In order to further confirm the prognostic value of miR-27a-3p and miR-379-5p, we then examined the expression levels of miR-27a-3p and miR-379-5p in pan-cancer by using CancerMIRNome online website. The results obtained reveal that miR-27a-3p and miR-379-5p were over-expressed in many cancer types, especially in COAD (**Figures 3E, F**). The number of tumors and normal samples, the area under the ROC curve (AUC), and the 95% confidence interval (CI) of the AUC for each cancer type in TCGA are shown in a forest plot, visualizing the results of the pan-cancer ROC analysis (**Figures 3G, H** and **Supplementary Figures 1H, I**). Therefore, these findings identified a robust ceRNA network that might correlate with prognosis in CRC patients.

### Identification of Prognosis Related 10 Key Genes in TCGA-COAD Cohort

**Figure 4:**
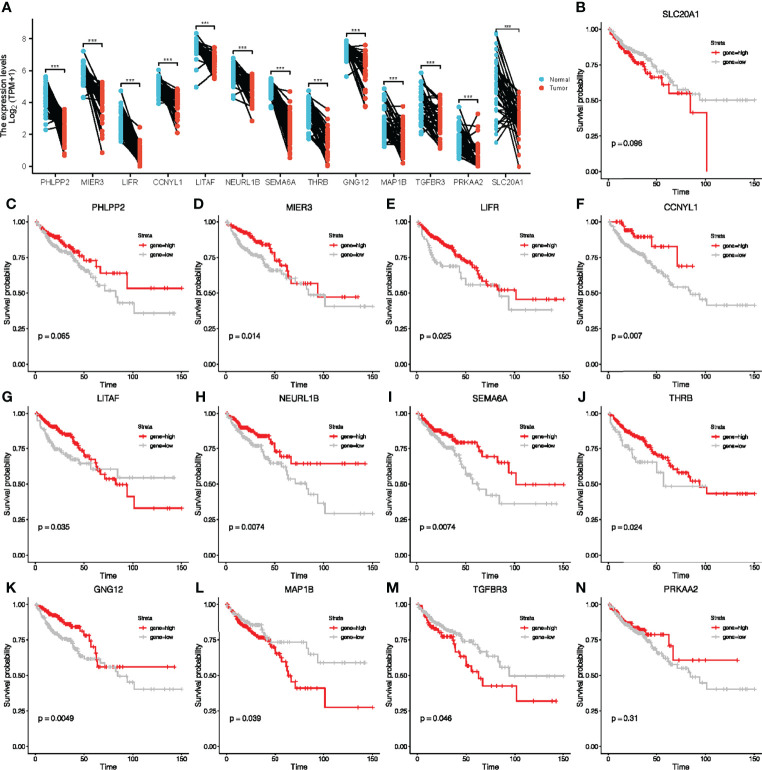
Paired sample expression analyses and survival analyses for 13 downstream genes. **(A)** The expression of target genes in TCGA-COAD. **(B–N)** Survival analyses were performed to further investigate the prognostic value of target genes based on the TCGA-COAD cohort.

To further explore the potential prognostic value in CRC of the ceRNA network, we first analyzed the expression levels of these target genes in tumor tissues. Our results showed that the expression of these target genes in tumor tissues was also significantly different from that in adjacent normal tissues (**Figure 4A**). Survival analyses were performed to further investigate the prognostic value of target genes based on the TCGA-COAD cohort. We found out that patients with high expressions of MIER3, LIFR, CCNYL1, LITAF, NEURL1B, SEMA6A, THRB, GNG12, MAP1B, and TGFBR3 had significant survival advantages over those with low expressions (**Figures 4B–N**). Thus, we selected these 10 key genes for further analysis of the value of the ceRNA network in clinical prognosis and immunotherapy.

### Pan-Cancer Analysis for Key Genes

**Figure 5:**
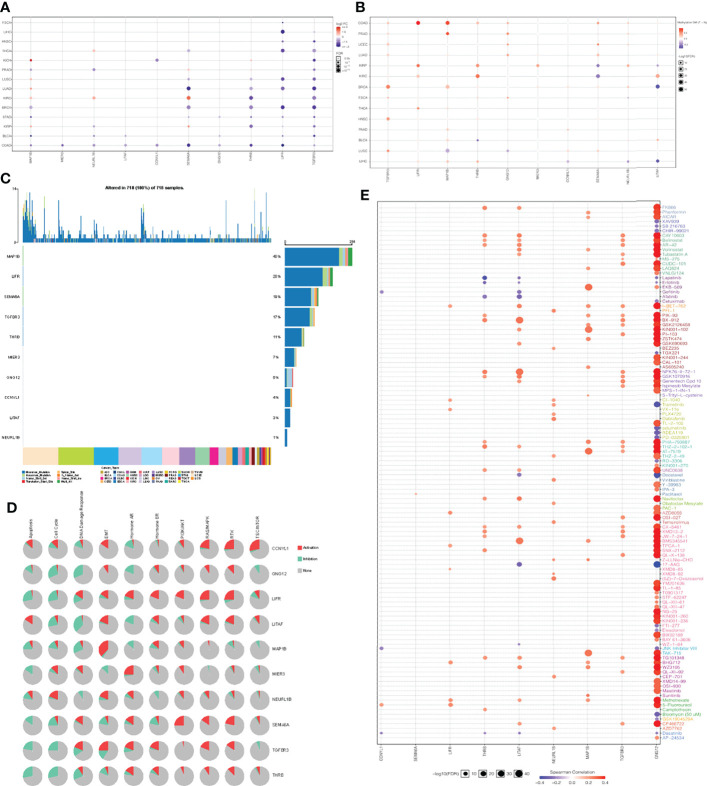
Pan-cancer analyses of 10 key genes based on GSCA dataset. **(A)** Key genes expression in pan-cancer. **(B)** Key genes methylation in pan-cancer. **(C)** Key genes mutation in pan-cancer. **(D)** Key genes related pathway in pan-cancer. **(E)** The correlation between gene expression and the sensitivity of GDSC drugs in pan-cancer.

Genomic changes in different cell types in different parts of the body lead to hundreds of different forms of cancer, resulting in tumors with diverse biological, pathological and therapeutic strategies (40). To explore the heterogeneity and commonality of these 10 genes in different cancers, we used GSCAlite website to perform some pan-cancer analyses for these significant genes. In most cancer, these genes are downregulated in expression and upregulated in methylation (**Figures 5A, B**). Considering that the occurrence and development of tumors were closely related to genetic mutations, we then looked at mutations in these key genes in pan-cancer. Among 718 samples, it was observed that MAP1B exhibited the highest mutation frequency followed by LIFR (**Figure 5C**). We noted that the types of variants in key genes were focused on missense mutations, suggesting that missense mutations may play a key role in the occurrence and progression of tumors. We then analyzed the enrichment of tumor-related pathways in 10 key genes (**Figure 5D**). These pathways have been widely confirmed to be involved in the proliferation, apoptosis, invasion and metastasis of cancer cells. Notably, all of these 10 genes except NEURL 1B showed a significant negative correlation in the cell cycle, and all of them except CCNYL1 and LITAF significantly inhibited the apoptosis of tumor cells. Moreover, the 10 genes also showed a good correlation in DNA damage repair. In order to explore the predictive effect of the has_circ_0066351-regulated ceRNA network in tumor immunotherapy, we assessed the drug sensitivity (IC50) of the expression levels of these 10 genes and found out that the higher the association, the higher the sensitivity. Notably, GNG12 showed a significant positive correlation with a variety of drugs (**Figure 5E**), which accord closely with the findings of the low expression associated with poor prognosis in osteosarcoma (41). Therefore, drug sensitivity analysis of the 10 genes network is helpful for personalized and precise treatment of tumor patients, reducing drug resistance. Taking the above results into consideration, it could be concluded that has_circ_0066351-regulated ceRNA network could be used as a biomarker of good prognosis in pan-cancer, and is closely related to tumor drug therapy, especially immunotherapy.

### Functional Characterization of Key Genes in Cancer Based on Single-Cell Data

**Figure 6:**
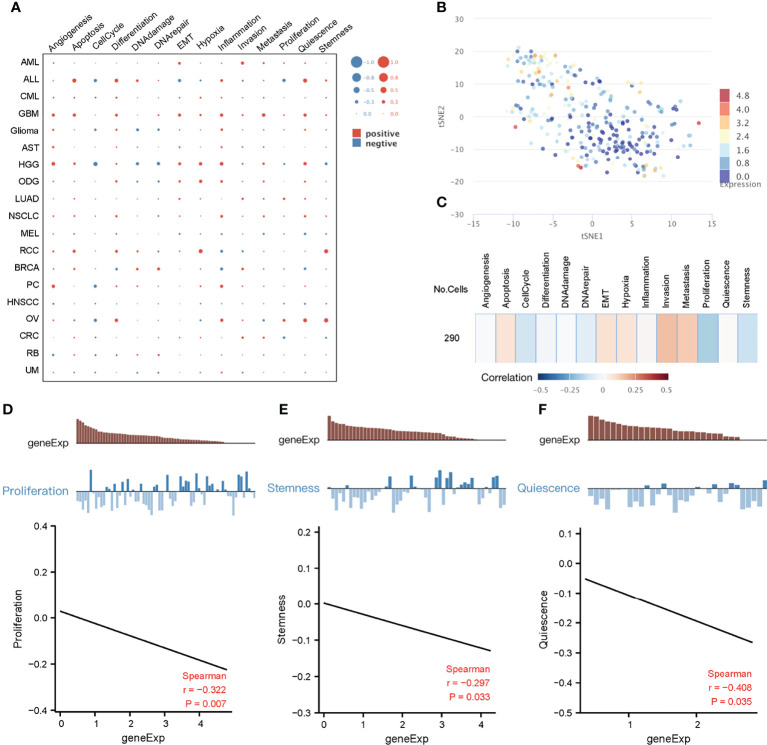
Functional relevance of key genes in cancers based on CancerSEA dataset. **(A)** The functional state of key genes across 19 types of cancer. The red plots indicated positive correlation while the blue plots indicated negative correlation. **(B)** T-SNE plot describes the distribution of cells, every point represents a single cell, and the color of the point represents the expression level of key genes in the cell. **(C)** Functional relevance of key genes in CRC. **(D)** Correlation between key genes expression and proliferation. **(E)** Correlation between key genes expression and stemness.

Due to cellular heterogeneity, the genetic information of cells with the same phenotype may differ significantly and much of the low abundance information may be lost in the overall characterization (42). In order to make up for the limitations of traditional high-throughput sequencing, single-cell sequencing technology emerged. To better understand the underlying functions and mechanisms of key genes in cancer, we used the CancerSEA database to study the functional status of 10 key genes in pan-cancer at the single-cell level (**Figure 6A**). To further explore the clinical application value of these 10 genes in colorectal cancer, we visualized the distribution of these genes in CRC at the single-cell level using T-SNE. Interestingly, analysis at the single-cell level showed that these 10 key genes were significantly downregulated in CRC. (**Figure 6B**). Single-cell enrichment analysis of tumor-related pathways revealed that these genes were negatively correlated with proliferation (**Figure 6C**). What’s more, functional correlation analysis confirmed that the expression of 10 key genes was negatively correlated with proliferation and stemness of CRC (**Figures 6D, E**), which further indicated that downregulation of these 10 genes, even has_circ_0066351-regulated ceRNA network, could be used as a biomarker of good prognosis of CRC.

### Multi-Omics Analyses and Immune Cell Infiltration Characterization of Identified Key Genes in COAD

**Figure 7:**
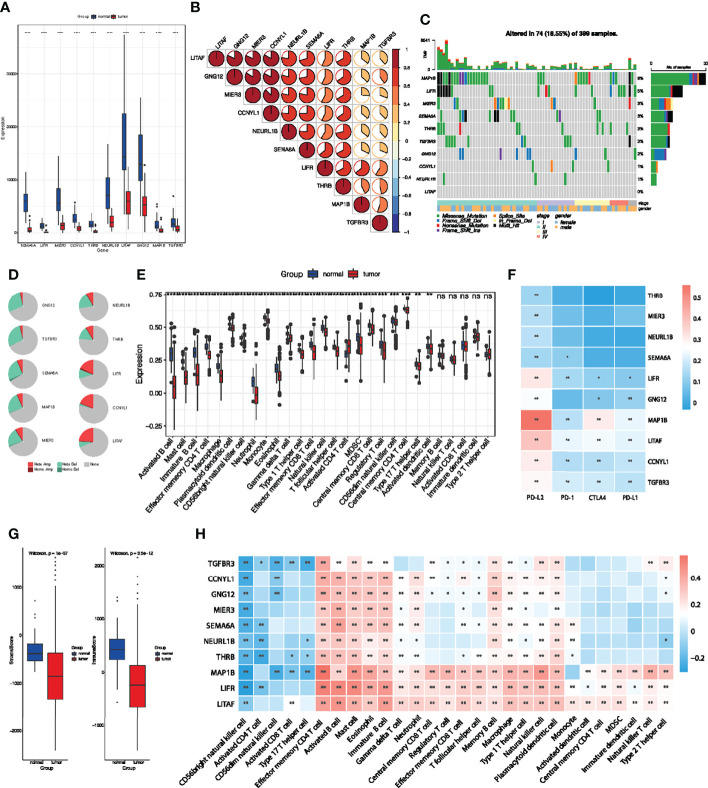
Multi-omics analyses of identified key genes and evaluation of TME immune cell infiltration characterization. **(A)** Validation of key genes expression in TCGA-COAD dataset. **(B)** The correlation between the 10 key genes using Spearman analyses. Negative correlation was marked with blue and positive correlation with red. **(C)** The mutation landscape of key genes in 399 samples of TCGA-COAD cohort. **(D)** CNV percentage in COAD patients. **(E)** Differences in 24 TME infiltration cells between normal and COAD tissues. **(F)** The correlation between the 10 genes and immune checkpoint molecules. **(G)** The difference of overall immune and stromal activity between normal and tumor tissues using ESTIMATE algorithm. **(H)** The correlation between each key gene and each TME infiltration cell type. Red, positive; Blue, negative. *P < 0.05; **P < 0.01; ***P < 0.001; ns, not significant.

Analysis of these identified 10 key genes in pan-cancer suggested that they were associated with good prognosis and immunotherapy sensitivity. To further clarify their value in colorectal cancer, we then focused on the role of key genes in COAD patients. The results obtained show that these 10 key genes were significantly downregulated in the TCGA-COAD cohort (**Figure 7A**). Spearman correlation analysis also showed that there were significant positive correlations among key genes (**Figure 7B**), which indicate that these key genes could influence and regulate each other. As mentioned above, these genes presented a rich mutation rate in pan-cancer, we then investigated the mutation landscape of key genes in COAD. Among 399 samples, 18.55% of patients experienced mutations in at least one key gene. We found out that MAP1B had the highest mutation frequency, followed by LIFR, while LITAF did not have any mutation in the COAD samples. Consistent with the pan-cancer analysis, the variant types of key genes mostly focused on the missense mutation and stage II (**Figure 7C**). The copy number variation (CNV) of the 10 genes is shown in **Figure 7D**. Among them, most key genes such as GNG12 TGFBR3, SEMA6A, MAP1B, MIER3, NEURL1B and THRB mutations were concentrated in the heterozygous amplification, while LIFR, CCNYL1 and LITAF were focused on the heterozygous deletion.

We then evaluated the landscape of 24 TME cell infiltration in normal and tumor samples to explore the role of 10 key genes in TME immune cell infiltration. As shown in **Figure 7E**, we found significant differences in the infiltration of 24 TME cells between normal and tumor samples. Among them, the infiltration of tumor-killing cells such as activated B cell, mast cell, immature B cell, macrophage, myeloid-derived suppressor cells (MDSCs), effector memory CD8^+^ T cell and neutrophils in tumor tissues were significantly lower than that in normal tissues (**Figure 7E**), which suggest that the patterns of TME immune cell infiltration may contribute to immune escape and resistance to immunotherapy. We also found out that the expression of the 10 key genes was positively correlated with immune checkpoint molecules, especially PD-L2 (**Figure 7F**). The results obtained show that the immunoactivity and interstitial activity of tumor tissue were significantly lower than that of normal colorectal cancer tissue (**Figure 7G**). To explore the internal association between key genes and TME infiltrating cells, we conducted a correlation analysis between key genes and TME infiltrating cells. Spearman correlation analysis revealed a significantly positive correlation between these genes and activated B cell, mast cell, immature B cell, macrophage, MDSCs, effector memory CD8^+^ T cell and neutrophils (**Figure 7H**), which exhibited lower infiltration in tumor tissue. These results suggest that deletion or mutation of key genes may lead to tumor occurrence and progression by reducing the infiltration of immune cells in TME.

### Construction of Prognostic and Immunotherapeutic Risk Model

**Figure 8:**
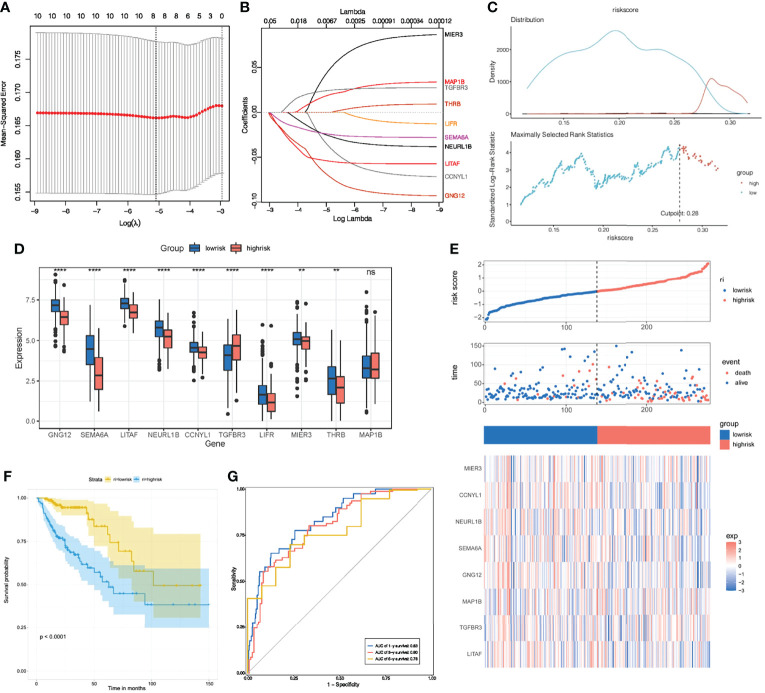
Construction of riskScore signature. **(A)** Tuning parameter selection by tenfold cross-validation in the LASSO model. **(B)** Least absolute shrinkage and selection operator (LASSO) coefficient profiles of the 10 key genes. **(C)** The optimal cut-off points to dichotomize riskScore into low and high groups was determined by MaxStat R package. The optimal cut-off point was 0.28. **(D)** The 10 key genes expressed in the low and high risk groups. **(E)** Proportion of deaths in high and low risk groups as riskScore values increased. Clustering of key genes between low and high risk groups. Red, up-regulated; Blue, downregulated. **(F)** Survival analyses for low and high riskScore groups using Kaplan-Meier curves. **(G)** Time-dependent ROC curves validation at 1, 3, and 5 years of prognostic value of the prognostic index in TCGA. *P < 0.05; **P < 0.01; ***P < 0.001; ns, not significant.

To further investigate the clinical value of these 10 key genes regulatory network, we conducted riskScore signature to integrate the roles of these 10 key genes through LASSO Cox regression model (**Figures 8A, B**). The MaxStat R package was used to determine the cutoff value 0.28 (**Figure 8C**), and the patients were divided into high and low risk groups. The expression of 10 key genes in tumors with low risk was significantly higher when compared to tumors with high risk (**Figure 8D**). As the risk value increased, patient mortality increased significantly (**Figure 8E**). Low risk patients showed better survival benefits (**Figure 8F**). Time-dependent analysis of subject operating characteristics was used to assess the predictability of the prognostic model. Our results showed that the AUC at 1, 3 and 5 years was 0.83, 0.80 and 0.78, respectively (**Figure 8G**). Therefore, we established a robust prognostic model based on key genes.

**Figure 9:**
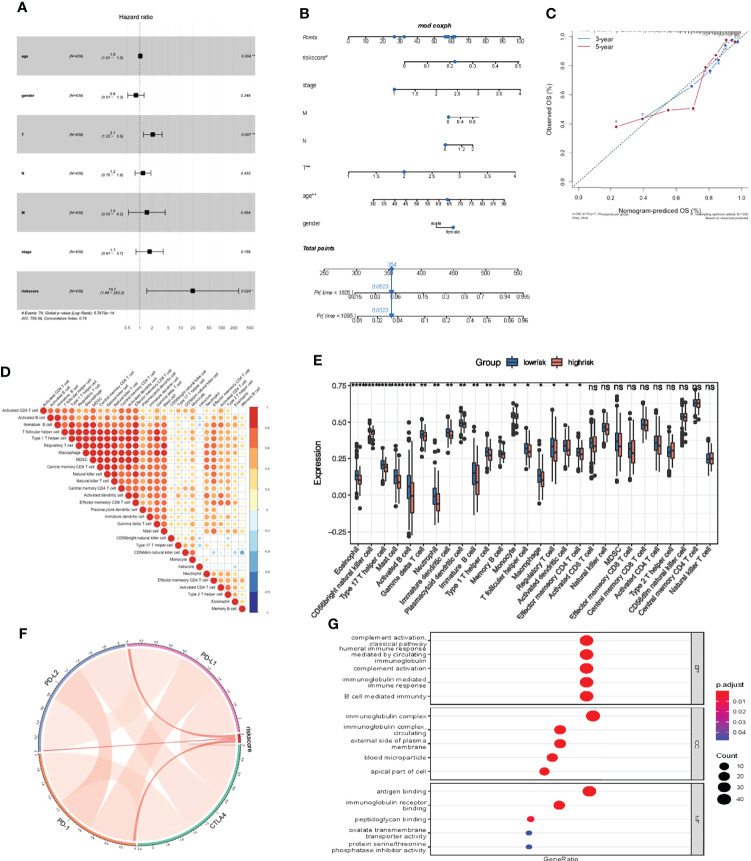
Prognostic and immune value of the riskScore gene signature. **(A)** Forest plot showing the riskScore was an independent prognostic biomarker using multivariate analyses. **(B)** The nomogram constructed to predict the probability of patient mortality. **(C)** The calibration plot of nomograms between predicted and observed 3-year and 5-year outcomes. The 45-degree line represented the ideal prediction. **(D)** The correlation between riskScore signature and 24 TME cell infiltration. Blue, negative correlation; Red, positive correlation. **(E)** Differences in 24 TME infiltration cells between low and high risk groups. **(F)** The correlation between riskScore signature and immune checkpoint molecules. Blue, negative correlation; Red, positive correlation. **(G)** The GO enrichment revealed the immune response pathway significantly activated in cancer.

Multivariate Cox regression model analysis included age, gender, clinical stage, T, M and N stage, indicating that riskScore characteristics can be used as a robust and independent prognostic biomarker to evaluate the prognosis of COAD patients (**Figure 9A**). To establish a clinically relevant method for quantitatively predicting the probability of patient death, we established a nomogram risk map that combined riskScore and independent clinical prognostic factors. The calibration diagram showed that when compared with the ideal model, the derived nomogram had better performance (**Figures 9B, C**).

We then studied the differences in TME cell infiltration between the low risk and high risk groups. We performed a correlation analysis to reveal the potential relationship between riskScore signatures and each TME cell type (**Figure 9D**). The results obtained also show significantly increased infiltration of regulatory T cells in the high risk group, while other cell types increased in the low risk group (**Figure 9E**). We also found a significant positive correlation between riskScore values and the expression of immune checkpoint molecules, indicating that riskScore characteristics may play a role in predicting clinical responses to immunotherapy (**Figure 9F**). As shown in **Figure 9G**, these downregulated genes are obviously related to immunobiological processes, such as complement activation pathway, immunoglobulin mediated immune response, B cell mediated immunity, humoral immune response. All of the above results indicate that the low risk group had a better immune response, further suggesting the biomarker value of has_circ_0066351-regulated ceRNA network in colorectal cancer prognosis and immunotherapy.

## Discussion

In recent years, with the continuous progression of sequencing technology, increasing circRNAs have appeared in the view of scientists. Accumulating evidence of circRNAs biogenesis and function have provided new insights into the pathogenesis of many diseases, especially cancer (43). Its atypical function in regulating the proliferation, migration, invasion and metastasis of cancer cells makes it a potential biomarker and therapeutic target (44, 45). Abnormal regulation of circRNAs has also been found to be involved in drug resistance in clinical cancer therapies, including chemotherapy, targeted therapy and immunotherapy (46). In addition, we should pay more attention to drug-resistant circRNAs as markers of colorectal cancer recurrence risk (47). Colorectal cancer is a complex gastrointestinal malignancy caused by multiple gene mutations and signaling pathway disturbances (48–50). However, the molecular mechanism of circRNAs in CRC remains unclear. In this study, transcriptome sequencing and bioinformatics analysis were performed to screen out abnormally regulated circRNAs and prove that hsa_circ_0066351 is a significantly downregulated key circRNAs in CRC tissues. Low hsa_circ_0066351 expression is closely related to distant metastasis, poor prognosis, immune cell infiltration and drug resistance.

To explore the biological function of circRNAs as biomarkers in CRC, we first characterized the circRNAs profile in human colorectal cancer using circRNA-seq analysis from three pairs of human CRC and adjacent non-tumor tissues. After bioinformatics analysis and pre-experiment, we finally selected hsa_circ_0066351 for follow-up research. RT-qPCR was used to detect the expression of hsa_circ_0066351 in our own colorectal cancer sample library and it was found that the expression of hsa_circ_0066351 was down-regulated in CRC tissues when compared with adjacent normal tissues, suggesting that hsa_circ_0066351 may have tumor-suppressive effect. In a further functional study, hsa_circ_0066351 significantly facilitated the proliferation of CRC cells.

Inhibition of miRNAs is considered to be the main function of circRNAs, mainly because circRNAs are more stable than linear mRNAs and long non-coding RNAs (39). We predicted that miRNAs is bound to hsa_circ_0066351 through multiple miRNA online prediction website and intersected with downregulated miRNAs in cancer in TCGA database to obtain two miRNAs: miR-27a-3p and miR-379-5p. Interestingly, it has been reported that miR-27a-3p was absorbed by circRNA sponge and lost its carcinogenic function (51). Our results also showed that miR-27a-3p and miR-379-5p were upregulated in most cancers. Taken together, these evidences further proved that hsa_circ_0066351 can inhibit cancer through sponge adsorption to miRNAs.

After predicting the interactions between circRNA, miRNA and mRNA and their effects on tumor biological behavior, we constructed a circRNA-miRNA-mRNA network. Then, according to prognosis analysis, the following 10 genes were identified as central downstream genes: MIER3, LIFR, CCNYL1, LITAF, NEURL1B, SEMA6A, THRB, GNG12, MAP1B and TGFBR3. Most of these 10 key genes have been shown to be downregulated in CRC and associated with a good prognosis. Among them, MIER3 could inhibit the progression of CRC by inhibiting epithelial-mesenchymal transformation (52). LITAF was identified to be a downstream target of AMPK that inhibited tumor growth (53). NEURL1B was regulated by miRNAs and served as a diagnostic and prognostic target for CRC (54). Low GNG12 expression was associated with a poor prognosis of osteosarcoma (41). Furthermore, exosome-derived microRNA-424 inhibited the growth of CRC by up-regulating TGFBR3 (55). These results provided a robust basis for integrating the has_circ_0066351-regulated ceRNA network based on these 10 key genes. Although most of these 10 key genes, such as MIER3, LITAF, NEURL1B, SEMA6A, GNG12 and TGFBR3, have been confirmed to be downregulated in CRC and inhibit the progression of CRC. However, there are still four genes LIFR, CCNYL1, THRB and MAP1B that lack sufficient evidence to prove their role in CRC.

To further explore how these 10 key genes were propagated in the biological behavior of the tumor, we looked at the mutation rate of the 10 genes in pan-cancer and found out that: in 718 samples, MAP1B showed the highest mutation frequency. At the same time, we also noted that the mutation types of these 10 key genes were mainly missense mutations, which suggested that further study on the types of these missense mutations and their role in inhibiting tumor progression was needed. More significantly, these 10 key target genes were closely related to tumor drug resistance. This suggests that further study of these 10 key target genes could reduce the occurrence of tumor drug resistance. Functional correlation analysis by single-cell sequencing database further confirmed that the expression of these 10 key genes was negatively correlated with proliferation and stemness of colorectal cancer.

In order to analyze the tumor biological behavior of these 10 downstream genes of hsa_circ_0066351, we focused on COAD for validation. We found out that the 10 key genes in COAD also showed excellent tumor-suppressive effect. Interestingly, just as in pan-cancer, missense mutation was the dominant mutation type of key genes in COAD, and MAP1B also showed the highest missense mutation frequency. Similarly, Mathewos et al. found out that reduced expression of MAP1B may make it prone to methylation, which may contribute to lung cancer (56). These provided a possible direction for future research on MAP1B: to explore the key role of missense mutations in the tumorigenesis and progression of cancer, especially colorectal cancer. Subsequently, we focused on the relationship between these 10 key genes and TME infiltrating cells in tumor tissues. We found out that immune cells with low infiltrating ability in TME were significantly positively correlated with the 10 key genes, which suggests that the key genes may exert their antitumor effects by enhancing the infiltration of immune cells in TME. Finally, the expression of these 10 key genes in high-risk tumors was significantly lower than that in low-risk tumors by dividing tumor patients into low-risk groups according to the regulatory network of these 10 key genes to build a prognostic model. Not only that, the low risk group in the prognostic model showed better survival. The AUC results also confirmed the reliability and authenticity of our model. Similarly, we applied the model to COAD cohort patients and found similar results: patients in the low-risk group showed better immune cell infiltration and immunotherapeutic effects. In the future, we can explore how to improve the immune cell infiltration in TME by targeting these key genes regulatory network to improve the efficacy of tumor immunotherapy (57).

Although the circRNA-miRNA-mRNA network appears to be a potential prognostic biomarker in clinical applications, several limitations must be noted. Firstly, the binding affinities of circRNA, miRNAs and mRNAs obtained from the database should be further investigated experimentally, such as luciferase or RNA pull-down. Secondly, the ceRNA network needs to be further validated by which signaling pathway it functions in tumors, particularly CRC. In addition, the further investigations of the function and mechanism of the key genes in CRC through experiments are needed. All in all, our results lay the foundation for further research in the future researches.

In summary, we identified 10 downstream key target genes by identifying a circRNA that was significantly downregulated in CRC and constructed a circRNA-miRNA-mRNA network. The prognostic model constructed by these 10 key target genes was found to have potential clinical application value by analyzing the prognosis, infiltration of immune cells and immunotherapy efficacy of these 10 key target genes in the cohort of pan-cancer and COAD. Further research is needed to elucidate the functional behavior of this regulatory network and prognostic model. Targeting this regulatory network in future research may alter the infiltration of adverse infiltration immune cells in TME as well as contribute to the development of new combination therapy strategies or new immunotherapy drugs.

## Data Availability Statement

The data presented in the study are deposited in the GEO repository, accession number GSE205643. All other data is available from the author upon reasonable request.

## Ethics Statement

This study was approved by Xiangya Hospital of Central South University. The patients provided their written informed consent to participate in this study.

## Author Contributions

YG, YZ, CC and SZ designed the study. YG, YZ, CC, SZ, LW, ZF, YC, PL, YP, LG, YL and YH collected the data and performed the major analysis. CC and SZ supervised the study. YG and YZ analyzed and interpreted the data. LW and ZF did the statistical analysis. YG, YZ and YH drafted the manuscript. All authors contributed to the article and approved the submitted version.

## Funding

This study was supported by grants from the National Key R & D Program of China (No. 2018YFC1313300), National Natural Science Foundation of China (No. 81772627, 81874073, 81974384, and 82173342), two projects from the Nature Science Foundation of Hunan Province (No. 2021JJ31092 and 2021JJ31048), and two projects from CSCO Cancer Research Foundation (No. Y-HR2019-0182 and Y-2019Genecast-043).

## Conflict of Interest

The authors declare that the research was conducted in the absence of any commercial or financial relationships that could be construed as a potential conflict of interest.

## Publisher’s Note

All claims expressed in this article are solely those of the authors and do not necessarily represent those of their affiliated organizations, or those of the publisher, the editors and the reviewers. Any product that may be evaluated in this article, or claim that may be made by its manufacturer, is not guaranteed or endorsed by the publisher.

## Supplementary Material

Supplementary Figure 1 Identified 2 miRNAs and 13 mRNAs as hsa_circ_0066351 regulated ceRNA network. **(A)** Combined with differentially expressed miRNAs upregulated by TCGA, three overlapping miRNAs were screened out: **(B)** hsa-miR-27a-3p, **(C)** hsa-miR-27b-3p, **(D)** hsa-miR-379-5p. **(E, F)** Combined with TCGA to downregulate differentially expressed mRNA, 13 target genes were screened. **(G)** The possible binding sites of has_circ_0066351 with miR-27a-3p and miR-379-5p. **(H, I)** Forest plot visualizing miR-27a-3p and miR-379-5p in pan-cancer AUC analysis. **P* < 0.05; ***P* < 0.01; ****P* < 0.001; ns, not significant.
