# Supplementary figures and images for: Hsa_Circ_0066351 Acts as a Prognostic and Immunotherapeutic Biomarker in Colorectal Cancer
Source: Front Immunol. 2022 Jul 13;13:927811. doi: 10.3389/fimmu.2022.927811 (PMC9667793; doi:10.3389/fimmu.2022.927811)

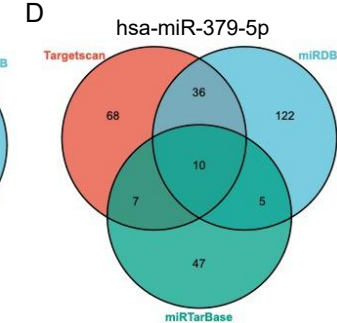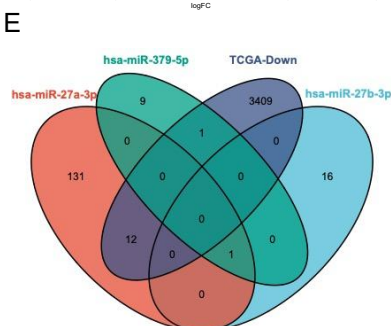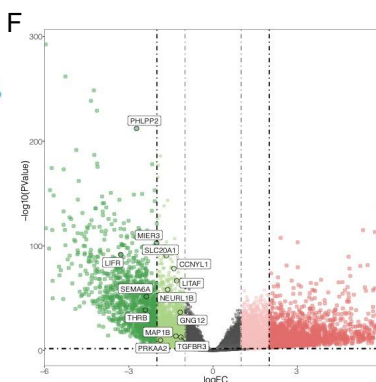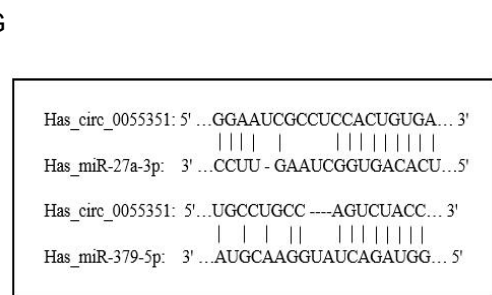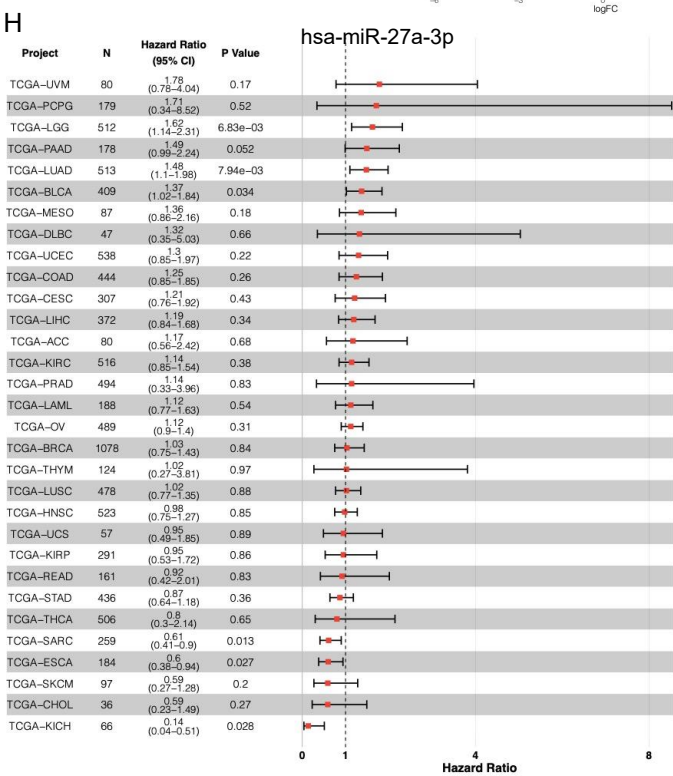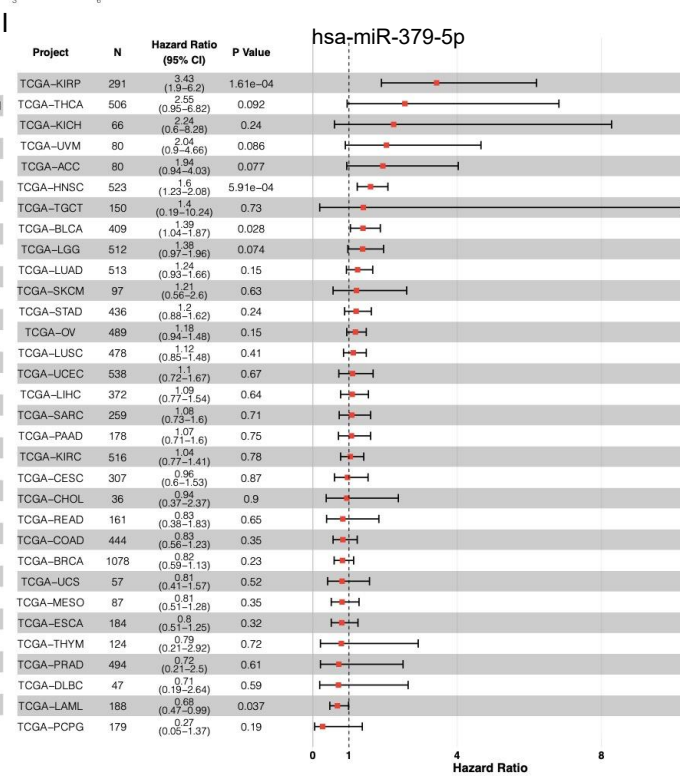

Supplement: Supplementary Figure 1 — Identified 2 miRNAs and 13 mRNAs as hsa_circ_0066351 regulated ceRNA network. (A) Combined with differentially expressed miRNAs upregulated by TCGA, three overlapping miRNAs were screened out: (B) hsa-miR-27a-3p, (C) hsa-miR-27b-3p, (D) hsa-miR-379-5p. (E, F) Combined with TCGA to downregulate differentially expressed mRNA, 13 target genes were screened. (G) The possible binding sites of has_circ_0066351 with miR-27a-3p and miR-379-5p. (H, I) Forest plot visualizing miR-27a-3p and miR-379-5p in pan-cancer AUC analysis. *P < 0.05; **P < 0.01; ***P < 0.001; ns, not significant. [file DataSheet_1.pdf]
